# Sialidase Deficiency in *Porphyromonas gingivalis* Increases IL-12 Secretion in Stimulated Macrophages Through Regulation of CR3, IncRNA GAS5 and miR-21

**DOI:** 10.3389/fcimb.2018.00100

**Published:** 2018-04-05

**Authors:** Xue Yang, Yaping Pan, Xiaoyu Xu, Tong Tong, Shiwen Yu, Yue Zhao, Li Lin, Jingbo Liu, Dongmei Zhang, Chen Li

**Affiliations:** ^1^Department of Periodontics, School of Stomatology, China Medical University, Shenyang, China; ^2^Shenyang Medical College, Shenyang, China; ^3^Department of Periodontics, Dalian Stomatology Hospital, Dalian Shi, China; ^4^Liaoning Province Key Laboratory of Oral Diseases, Shenyang, China; ^5^Liaoning Province Translational Medicine Research Center of Oral Diseases, Shenyang, China

**Keywords:** *P. gingivalis*, neuraminidase, macrophage, interleukin-12, microRNA-21, lncRNA *GAS5*

## Abstract

*Porphyromonas gingivalis* (*P. gingivalis*) is a major periodontal pathogen that can induce an immune response leading to a destructive inflammatory process. During the inflammatory process, interleukin-12 (IL-12) is secreted, correlating with bacterial clearance by macrophages. Bacterial sialidase has recently been shown to influence the synthesis and modification of the macromolecules on its surface, and is associated with the interaction between bacteria and host cells. We have previously constructed a *P. gingivalis* sialidase gene mutant strain in *P. gingivalis* W83 (ΔPG0352) and found that ΔPG0352 showed less pathogenicity than the wild-type strain. In this study, U937-differentiated macrophages were stimulated by *P. gingivalis* W83, ΔPG0352, or PG0352 complemented strain (comΔPG0352). Transmission electron microscopy showed that *P. gingivalis* caused a loss of membrane integrity in macrophages and the intracellular bacteria were enclosed within endocytic vacuoles. The expression of both *IL-12p35* and *IL-12p40* genes and the levels of IL-12p70 were significantly higher in U937 stimulated by ΔPG0352 than in those with *P. gingivalis* W83 and comΔPG0352. In order to explain why ΔPG0352 induced more IL-12 in macrophages, immunofluorescence assays, PCR arrays, and gene silence or overexpression experiments were carried out. Immunofluorescence assays showed that ΔPG0352 induced lower expression of CR3 in macrophages. After CR3 was suppressed, there were no significant differences in the IL-12p70 levels between macrophages stimulated by *P. gingivalis* W83, ΔPG0352 or comΔPG0352. PCR array experiments showed that miR-21 and lncRNA *GAS5* were differentially expressed between macrophages stimulated by *P. gingivalis* W83 and ΔPG0352, which had been identified by real-time PCR. The results of CR3 blocking and lncRNA *GAS5* gene silence or overexpression showed that the difference in IL-12 levels between *P. gingivalis* W83 and ΔPG0352 groups was associated with CR3, lncRNA *GAS5* and miR-21. Thus it can be concluded that the sialidase-deficient strain is more easily cleared by attenuating CR3 activation, reducing the inhibition of lncRNA *GAS5*, inducing less miR-21 and more IL-12 in macrophages. These results indicate that inhibiting the activity of sialidase in *P. gingivalis* will cause rapid clearing by macrophages.

## Introduction

Chronic periodontitis is a type of chronic inflammatory disorder of bacterial origin that affects tooth-supporting tissue, eventually leading to tooth loss (Highfield, 2009). Plaque is the initiator of chronic periodontitis caused by more than 700 microorganism species. Among these, *Porphyromonas gingivalis* (*P. gingivalis*) has been implicated as a major etiologic agent in the pathogenesis of chronic periodontitis (Loesche and Grossman, 2001; Aas et al., 2005). *P. gingivalis* is a Gram-negative an aerobic coccobacillus and can produce a number of virulence factors, such as fimbriae, gingipains and lipopolysaccharide (LPS) (Bostanci and Belibasakis, 2012). High levels of *P. gingivalis* induce an immune response leading to a destructive inflammatory process (Garlet, 2010). It is noteworthy that *P. gingivalis* can escape the host's innate immune response and internalize to the host cells, as well as invade into deep tissue (Lamont et al., 1995).

During the initiation and progression of periodontal disease, macrophages in the periodontal tissue play a central role in the front-line immune response against invading pathogens (Zhou et al., 2005). In response to infection by periodontal pathogens, macrophages eliminate pathogens by phagocytosis, or secrete cytokines leading to an inflammatory process (Reichrath et al., 2016). Macrophages also have antigen-presenting functions resulting in activation of acquired immunity (Lavin et al., 2015). During the inflammatory process, many cytokines and chemokines produced by macrophages activate the immune response (Okada and Murakami, 1998; Park et al., 2014). IL-12 is a heterodimeric proinflammatory cytokine, composed of the disulfide-linked p35 and p40 subunits to form bioactive IL-12 (also known as IL-12p70; Gately et al., 1998). IL-12p70 is correlated with bacterial clearance by macrophages (Trinchieri, 2003) and has been shown to stimulate NK cell production. In addition, IL-12p70 promotes CD4^+^ T cell differentiated from Th1 cells to Th2 cells, and can also activate the bacterial clearance function of macrophages (Yun et al., 2001; Trinchieri, 2003).

The study of sialic acid and the sialidase gene in pathogenic bacteria has been the subject of much recent research. Sialic acids are nine-carbon sugar acids derived from *N*-acetylneuraminic acid (Vanterpool et al., 2006). Their presence or absence has played an important role in determining the physiological state of a cell or tissue. Many symbiotic pathogens take advantage of sialic acids in their environment (the host) as sources of carbon, nitrogen, energy, and amino acids to synthesize the cell wall (Plumbridge and Vimr, 1999). Sialic acids may also regulate the expression of other genes, such as the fimbriae gene, and may modify macromolecules on the surface of bacteria (El-Labany et al., 2003; Comstock and Kasper, 2006; Severi et al., 2007). Sialylation on the surface of bacteria on the one hand enhances the virulence of bacteria; while on the other hand leads to the host mistakenly recognizing the pathogen as host cells evading an immune response. Such a mechanism is beneficial to the pathogen's ability to escape the host's defense. There are two ways for bacteria to acquire sialic acid: endogenous synthesis and exogenous decomposition (Severi et al., 2007). Endogenous synthesis occurs in bacteria that contain enzymes associated with sialic acid synthesis. While some bacteria, such as *P. gingivalis*, do not contain these enzymes and cannot synthesize sialic acid; instead, they obtain sialic acid from the environment by sialidase in a process known as exogenous decomposition. Sialidase (neuraminidase) is a type of glycosyl hydrolase that can cut the connection between glycosylation and the sialic acid O-receptor substrate by external hydrolysis to obtain free sialic acid (Vimr, 1994). The sialidase gene can be expressed in a variety of pathogens, is associated with macromolecule synthesis and modification, leading to infection, tissue damage, peroxide elimination in the process of oxidative stress and host innate immune regulation (Vimr, 1994; Iijima et al., 2004; Wang et al., 2010).

As early as 1990, Moncla et al. discovered that *P. gingivalis* showed sialidase activity (Moncla et al., 1990). However, the roles of sialidase in *P. gingivalis* have not been studied further possibly due to limitations in technology and knowledge. Recently, we have constructed a mutant strain of the *P. gingivalis* sialidase gene in *P. gingivalis* W83 (ΔPG0352) by means of homologous recombination. It was found that *PG0352* was the sole gene encoding sialidase in *P. gingivalis* W83. Sialidase deficiency did not influence *P. gingivalis* growth, but showed less pathogenicity than the wild-type strain in a mouse abscess model (Li et al., 2012), while the mechanism remains unknown. Our previous study and the study of Aruni W et al. showed that the existence of sialidase was associated with the expression of virulence factors in *P. gingivalis*, including fimbriae, gingipain, capsule, and LPS (Aruni et al., 2011; Li et al., 2017). The sialylation on the surface of pathogen made it difficult to be recognized by macrophages, allowing escape from the hosts' defense. Therefore, we hypothesized that sialidase deficiency prevented *P. gingivalis* from acquiring sialic acid, thus affecting the virulence factors and modification of macromolecules. As a result, sialidase-deficient *P. gingivalis* is easier to be recognized and activate phagocytosis in macrophages.

In this study, we stimulated U937-differentiated macrophages with *P. gingivalis* W83 and ΔPG0352, compared the differences in IL-12 expression, and analyzed the differences of miRNAs and lncRNAs by PCR array. In addition, gene silence or overexpression experiments were conducted on the lncRNA *GAS5* in order to decipher the mechanism of the effect of sialidase on *P. gingivalis* subverting phagocytosis by macrophages.

## Materials and methods

### Bacterial strains and growth conditions

The construction of ΔPG0352 and comΔPG0352 were as previously described (Li et al., 2017). *Porphyromonas gingivalis* W83, ΔPG0352 and comΔPG0352 were cultured on tryptic soy blood (TSB) agar plates supplemented with 5% sheep blood, 5% yeast extract, hemin (5 μg/ml) and 0.1% vitamin K under anaerobic conditions at 37°C. Bacterial suspensions were prepared in TSB infusion broth at 37°C under anaerobic conditions (N_2_, 80%; H_2_, 10%; CO_2_, 10%).

### Cell culture and macrophage preparation

Human monocytes U937 (ATCC CRL-1593.2), a monoblastic leukemia cell line were purchased from American Type Culture Collection (Manassas, VA, USA). The cells were cultured in RPMI1640 supplemented with 10% heat-inactivated fetal bovine serum (FBS; Gibco, USA) and antibiotics (100 units/ml penicillin, 100 μg/ml streptomycin, Sigma, USA) at 37°C, 5%CO_2_. The cells were then incubated with 10 ng/ml phorbol myristic acid (PMA; Sigma, USA) to induce differentiation into macrophage-like cells. After 72 h, the medium was replaced with fresh medium without PMA or antibiotics, and the differentiated cells were incubated for an additional 24 h prior to use (Roberts et al., 1997).

### MTS assay

Cell proliferation assays were performed using the CellTiter 96® Aqueous One Solution Cell Proliferation Assay kit (Promega, Madison, WI, USA) according to the manufacturer's instructions. In brief, U937 cells (1 × 10^4^ cells/well) were seeded in 24-cell culture plates with PMA. After differentiation into macrophage-like cells, the cells were stimulated by *P. gingivalis* W83, ΔPG0352 or comΔPG0352 (MOI = 100) for 0, 2, 6, 8, 12, 24, and 48 h. A total of 20 μl MTS reagent was added into each well at each time point and incubated for 4 h, and finally the absorbance at 490 nm monitored using a microplate reader.

### Transmission electron microscopy (TEM)

U937-differentiated macrophages were stimulated by *P. gingivalis* W83, ΔPG0352, or comΔPG0352 (MOI = 100) for 6 h at 37°C, 5%CO_2_. TEM was performed according to methods previously described (Giacona et al., 2004; Willingham et al., 2007). Briefly, cells were washed, scraped and centrifuged at 800 rpm for 5 min. The pellets were fixed in 2.5% glutaraldehyde at 4°C overnight. Then, cells were washed three times with sterilized water and suspended in osmium solution for 2 h. The washed cell pellets were dehydrated in a graded of series of ethanol and acetone solutions (30, 50, 70, and 80%, 90 and 100% respectively for 30 min each). Finally, the pellets were embedded in Epox 812 resin. Thin sections were cut, post-stained with uranyl acetate and lead citrate, and viewed using the transmission electron microscope (H-7650, HIACHI, Japan).

### Real-time polymerase chain reaction (real-time PCR) for *IL-12p35* and *IL-12p40*

U937-differentiated macrophages were stimulated by *P. gingivalis* W83, ΔPG0352 or comΔPG0352 (MOI = 100) for 6, 12, and 24 h at 37°C, 5%CO_2_. Total RNA was extracted from cells (miRNeasy Mini Kit, Qiagen, Germany) and reverse-transcribed (PrimeScript™ RT Master Mix kit, Takara, Japan) according to the manufacturer's instructions. Real-time PCR was performed with a SYBR Green approach (SYBR® Premix Ex Taq, Takara, Japan) and the expressions of *IL-12p35* and *IL-12p40* were measured with ABI System (Applied Biosystems, USA). The primers were as follows: *GAPDH*: F: 3′-GAAGGTGAAGGTCGGAGTC-5′ and R: 3′-GAAGATGGTGATGGGATTTC-5′ (Dutzan et al., 2012); *IL-12p35*: F: 3′-CACTCCCAAAACCTGCTGAG-5′ and R: 3′-TCTCTTCAGAAGTGCAAGGGTA-5′ (Farkas et al., 2008); *IL-12p40*: F: 3′-CCCTGACATTCTGCGTTCA-5′ and R: 3′-AGGTCTTGTCCGTGAAGACTCTA-5′ (Farkas et al., 2008). The amplification cycling conditions were 95°C for 30 s, 40 cycles of 5 s each at 95°C, and 34 s at 60°C. 2^−ΔΔ*Ct*^ was used to evaluate the variability of target genes as previously described (Suzuki et al., 2005).

### Enzyme-linked immunosorbent assay (ELISA)

U937-differented macrophages were cultured in 6-well culture plates and a final concentration of 2 μg/ml anti-CD11b antibody was added for pretreatment (1 h) in inhibitor groups. Then macrophages were stimulated by *P. gingivalis* W83, ΔPG0352 or comΔPG0352 (MOI = 100) for 6, 12 and 24 h. The levels of IL-12p70 were determined using a Human IL-12A/IL-12B ELISA kit (Sigma-Aldrich, USA), according to the manufacturer's instructions. A microplate reader was used to measure optical density at 450 nm. Standard curves were generated by plotting the mean optical density and concentration of each standard dilution, and the concentrations of each indicator in samples were calculated.

### Immunofluorescence assay

U937-differentiated macrophages cultivated on 24-well chambered cover-glass slides were stimulated by *P. gingivalis* W83, ΔPG0352 or comΔPG0352 (MOI = 100) for 6 h and were fixed in 4% paraformaldehyde at 4°C for 60–90 min. Cells were then punched with 0.25% Triton for 30 min and blocked in PBS with 10% human serum for 20 min. Samples were incubated with CR3 specific antibody CD11b (1:100 dilution in PBS solution; Abcam, USA) overnight at 4°C, and incubated with the secondary antibody conjugated to fluorescein isothiocyanate isomer (FITC) (1:100 dilution in PBS solution; Abcam, USA) for 30 min at 37°C. The slides were incubated with DiIC_18_ (3) (DiI) and 4,6-diamidino-phenylindole (DAPI) for 20 and 5 min respectively. Quencher was added and observed under a fluorescence microscope. Positive-staining sections in each cell of a ×200 image were analyzed for the intensity of fluorescence emitted with National Institutes of Health (NIH) IMAGEJ analysis software.

### PCR array

U937-differentiated macrophages were stimulated by *P. gingivalis* W83 or ΔPG0352 (MOI = 100) for 6 h at 37°C, 5%CO_2_. Total RNA was extracted from macrophages with TRIzol and purified using a miRNeasy Mini Kit (Qiagen, Germany). A total of 200 ng RNA was reverse-transcribed to cDNA with miScript®II RT Kit (Qiagen, Germany). Next, 200 μl RNase-free water was added to each 20 μl cDNA solution. The reaction mixture was prepared, including 1,375 μl 2 × QuantiTect SYBR Green PCR Master Mix, 275 μl 10 × miScript Universal Primer, 1,000 μl RNase-free water and 100 μl cDNA according to the manufacturer's instructions of RT^2^ miRNA PCR Array (MIHS-105, Qiagen, USA). Next, 25 μl per well reaction mixture was added to the 96-well plates. Plates were placed in the real-time cycler and the amplification cycling conditions were programmed as follow: 95°C for 15 min, 40 cycles of 15 s each at 94°C, 30 s at 55°C and 30 s at 70°C. Data were analyzed using miScript miRNA PCR Array Data Analysis Tool. *SNORD48* and *RNU6-2* were used as references. RNA from three independent cultures was analyzed. TargetScan (http://www.targetscan.org/vert_50/) and miRDB (http://mirdb.org/miRDB/download.html.) databases were used to predict IL-12 related miRNAs for further exploration of differently expressed miRNA between *P. gingivalis* W83 and ΔPG0352 groups.

### LncRNAs selection

LncRNAs *MEG3, GAS5, FASLG, BTG2, SPRY2*, and *TAGAP* that correlated with miR-21 were primarily screened by referring previous reports. U937-differentiated macrophages were stimulated by *P. gingivalis* W83 or ΔPG0352 (MOI = 100) for 6 h at 37°C, 5%CO_2_. Total RNA was extracted from macrophages by using TRIzol reagent (Invitrogen, Carlsbad, CA). Real-time PCR was used to detect the expression of different lncRNAs. The sequences were as follow: *MEG3*: F: 5′-ATCATCCGTCCACCTCCTTGTCTTC-3′, R: 5′-GTATGAGCA TAGCAAAGGTCAGGGC-3′ (Jia et al., 2014); *GAS5*: F: 5′-CTTCTGGGCTCAAGTGATCCT-3′, and R: 5′-TTGTGCCATGAGACTCCATCAG-3′ (Khalil et al., 2009); BTG2: F:5′-CATCATCAGCAGGGTGGC-3′, R: 5′-CCCAATGCGGTAGGACAC-3′ (Li et al., 2015); *SPRY2*: 5′-GAGTCGTCTCCAGCTCCGAAC-3′, 5′-AGCTCTGGCCTCCATCAGG-3′ (Ahn et al., 2015).

### Preparation of shRNA-expressing lentiviruses and cell infection

A targeted sequence designed to be homologous to lncRNA *GAS5* were cloned into the LV3 (H1/GFP&Puro) and LV5 (EF-1αF/GFP&Puro) lentiviral vectors (GenePharma, Shanghai, China) for *GAS5* gene silence and overexpression, respectively (See Images 1, 2 in Supplementary Material). These vectors were termed LV3-*GAS5*-Lentivirus and LV5-*GAS5*-Lentivirus, and the vectors without lncRNA *GAS5* gene were LV3NC-*GAS5*-Lentivirus and LV5NC-*GAS5*-Lentivirus. The sequences were as follow: LV3-*GAS5*-Lentivirus: 5′-CTTGCCTGGACCAGCTTAA-3′; LV3NC-*GAS5*-Lentivirus: 5′-TTCTCCGAACGTGTCACGT-3′. The construction of LV3 and LV5 lentiviral vectors was as previously described (Shen et al., 2017). Briefly, the restriction enzyme cutting sites were digested by special enzyme (*Not*I&*Bam*HI or *Bam*I&*Eco*RI). Then the vectors were recovered using DNA gel recovery kit and the amplified fragment was subjected to recombinant cloning. The ligation products were transferred to the competent cells, and the newly generated clones were verified by double enzyme digestion. The vectors were transfected into the 293T packaging cell line with RNAi-Mate. The supernatant was collected after infection to harvest the recombinant virus. U937-differentiated macrophages cells were infected with the virus (MOI = 100) with 5 μg/ml polybrene and non-transfected cells were used as a control. The cells were divided into five groups: control group, LV3NC (control for lncRNA GAS5 silence), LV3 (lncRNA GAS5 silence), LV5NC (control for lncRNA GAS5 overexpression), and LV5 (lncRNA GAS5 overexpression) groups. After 72 h infection, the expression of green fluorescent protein (GFP) was observed using a fluorescent microscope to assess the infection efficiency. The silence efficiency of *GAS5* was evaluated by real-time PCR.

U937-differentiated macrophages cells were seeded on 6-well culture plates post-lentiviral infection, and stimulated by *P. gingivalis* W83 or ΔPG0352 (MOI = 100) for 24 h at 37°C, 5%CO_2._ The levels of IL-12p70 were determined using a Human IL-12A/IL-12B ELISA kit (Sigma-Aldrich, USA).

### Confirmation of differently expressed miR-21 and lncRNA *GAS5*

U937-differentiated macrophages were stimulated by *P. gingivalis* W83, ΔPG0352 and comΔPG0352 (MOI = 100) respectively for 6 h, 12 and 24 h at 37°C, 5%CO_2_ condition. RNA and protein was extracted. Real-time PCR was used to detect the changes of miR-21 and lncRNA *GAS5* gene expressions. The primers were as followed: miR-21: F: 5′-AGTGGGGAGTAGCTTATCAGAC-3′, and R: 5′- CAACGGGGCATCAACATCA-3′ (Yu et al., 2012); U6: F: 5′- CTCGCTTCGGCA GCACA-3′, R: 5′- AACGCTTCACGAATT TGCGT-3′ (Jia et al., 2014).

### Real-time PCR for GAS5 after CR3 receptor was suppressed

U937-differented macrophages were cultured in 6-well culture plates and a final concentration of 2 μg/ml anti-CD11b antibody was added for pretreatment (1 h). Then macrophages were stimulated by *P. gingivalis* W83, ΔPG0352 or comΔPG0352 (MOI = 100) for 6, 12, and 24 h. The expression of lncRNA *GAS5* was detected by real-time PCR.

### Statistical analysis

One-way analysis of variance (ANOVA) was used to compare the differences between parameters in each group. Measurement data were represented as mean ± standard deviation. All tests were two-sided, with a significance level of 0.05. All statistical analyses were performed with statistical software.

## Results

### The morphology of macrophages after various *P. gingivalis* infections

**Figure 1:**
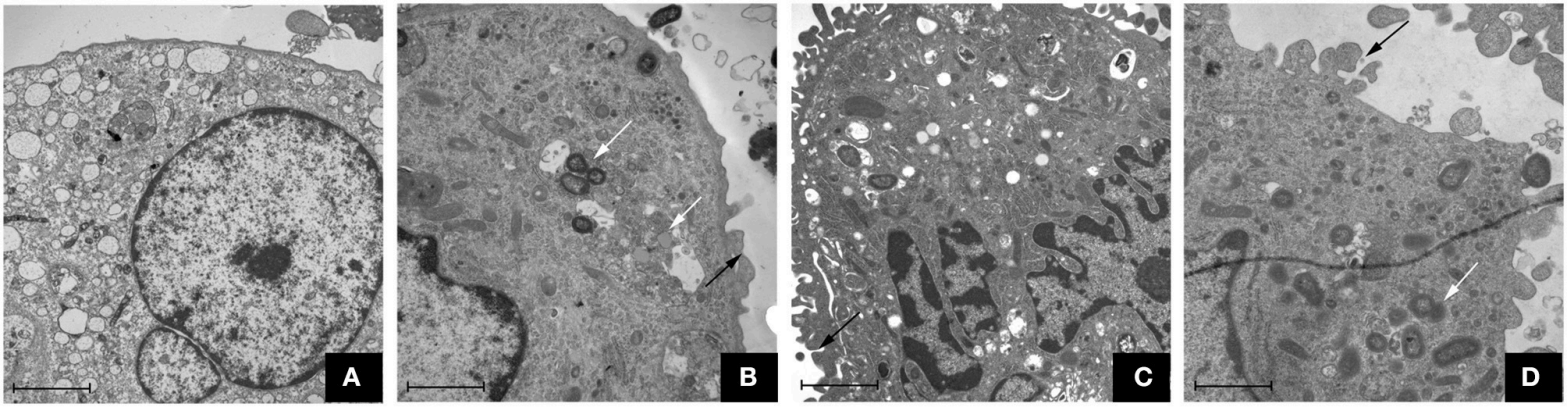
Transmission electron microscope observation of macrophages stimulated by *P. gingivalis* W83, ΔPG0352 and comΔPG0352 respectively (15,000 ×, bar: 2 μm). **(A)** control group; **(B)** *P. gingivalis* W83 group; **(C)** ΔPG0352 group; **(D)** comΔPG0352 group. White arrow: *P. gingivalis* internalized by macrophage; black arrow: *P. gingivalis* adhering to macrophage surface.

The TEM assay showed that the three *P. gingivalis* strains could induce uptake by macrophages. In the control group, macrophages were morphologically normal and the membrane was integrated (shown in Figure 1). However, there were microvilli and coated pits on the cell surface of macrophages in the *P. gingivalis* W83, ΔPG0352 and comΔPG0352 groups. The membrane was discontinuous and the intracellular bacteria were enclosed within endocytic vacuoles.

### Compared to *P. gingivalis* W83, ΔPG0352 induced more IL-12 in macrophages

**Figure 2:**
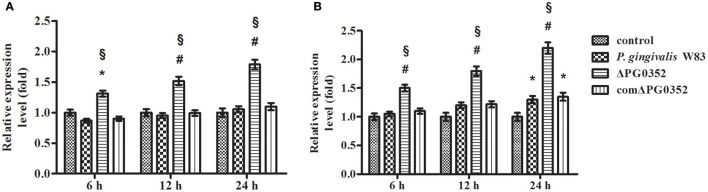
The expression of *IL-12p35* **(A)** and *IL-12p40* **(B)** genes after macrophages stimulated by *P. gingivalis* W83, ΔPG0352 or comΔPG0352. *P < 0.05 vs. control group, ^#^*P* < 0.01 vs. control group, ^§^*P* < 0.01 vs. *P. gingivalis* W83 group, using ANOVA.

**Figure 3:**
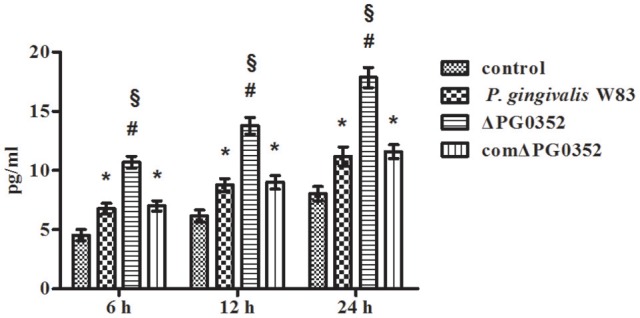
The levels of IL-12p70 after macrophages stimulated by *P. gingivalis* W83, ΔPG0352, or comΔPG0352. *P < 0.05 vs. control group, ^#^*P* < 0.01 vs. control group, ^§^*P* < 0.01 vs. *P. gingivalis* W83 group, using ANOVA.

The expression of *IL-12p35* and *IL-12p40* genes was detected by real-time PCR. Compared with the *P. gingivalis* W83 group, the expression of *IL-12p35* and *IL-12p40* genes were higher in the ΔPG0352 group (*P* < 0.01) (shown in Figure 2). The results of ELISA showed that, compared with the *P. gingivalis* W83 and comΔPG0352 groups, the IL-12p70 level was significantly higher in the ΔPG0352 group from 6 to 24 h (*P* < 0.01) (shown in Figure 3). There were no significant differences between the *P. gingivalis* W83 and comΔPG0352 groups in the gene expression of *IL-12p35* and *IL-12p40* and the protein level of IL-12p70 (*P* > 0.05).

### The difference in IL-12 level between *P. gingivalis* W83 and ΔPG0352 groups was associated with CR3

**Figure 4:**
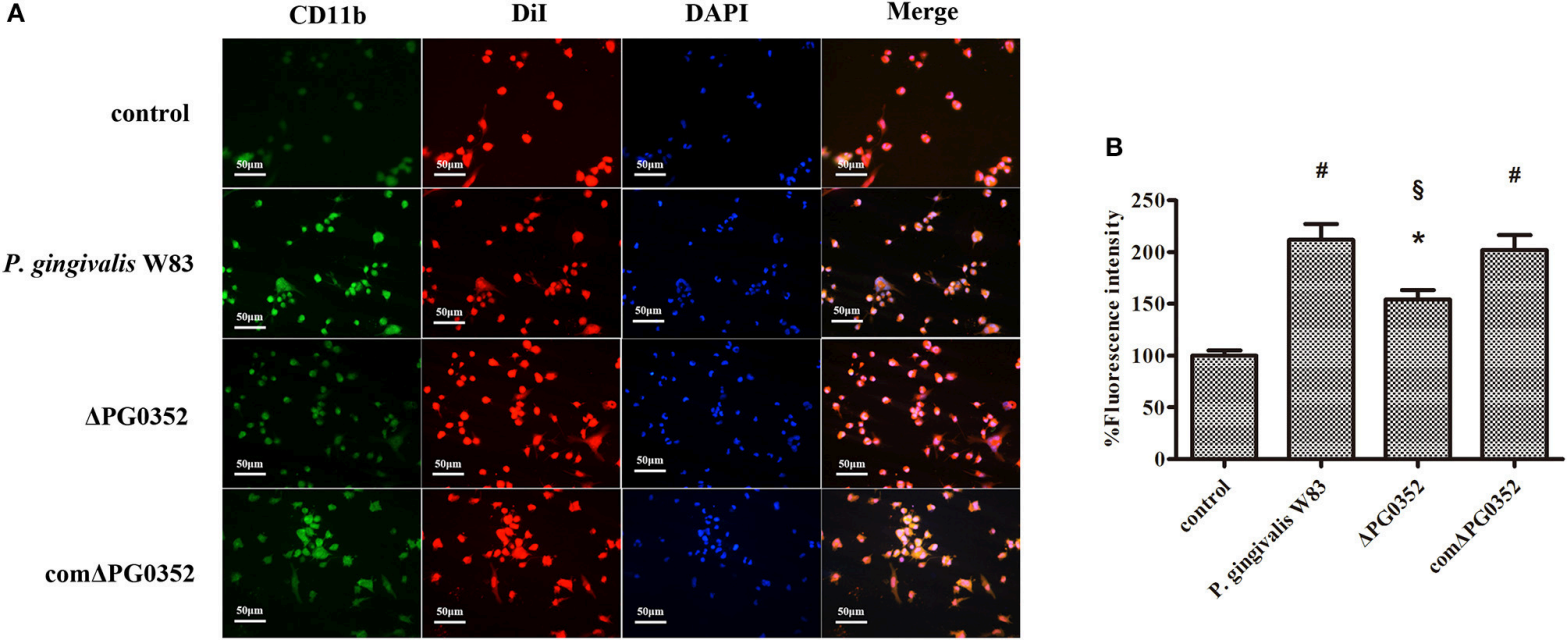
The CR3 expression levels after stimulated by *P. gingivalis* W83, ΔPG0352 and comΔPG0352. **(A)** The U937-differentiated macrophages membranes stained with DiI appear red, while the nuclei appeared blue (200×). **(B)** Analysis of fluorescent levels using IMAGEJ software revealed elevated CD11b levels in *P. gingivalis* W83, ΔPG0352 and comΔPG0352 groups compared with control group. After 6 h post-infection, the fluorescence intensity of CR3 in *P. gingivalis* W83 group was about 2 times higher than that in the control group, and about 1.5 times higher than that in ΔPG0352 group. There were no significant differences between *P. gingivalis* W83 and comΔPG0352 groups. *P < 0.05 vs. control group, ^#^*P* < 0.01 vs. control group, ^§^*P* < 0.01 vs. *P. gingivalis* W83 group, using ANOVA.

As shown in Figure 4A, CR3 was expressed on almost the entire cell surface, but the fluorescence intensity was different in each group. NIH software IMAGEJ was used to analyze the fluorescence intensity of CR3 expression in each group. The fluorescence intensity levels in the *P. gingivalis* W83, ΔPG0352 and comΔPG0352 groups were significantly higher than that in the control group (shown in Figure 4B). The fluorescence intensity of CR3 in the *P. gingivalis* W83 group was about two times higher than that in the control group, and about one and a half times higher than that in the ΔPG0352 group. There were no significant differences between the *P. gingivalis* W83 and comΔPG0352 groups.

**Figure 5:**
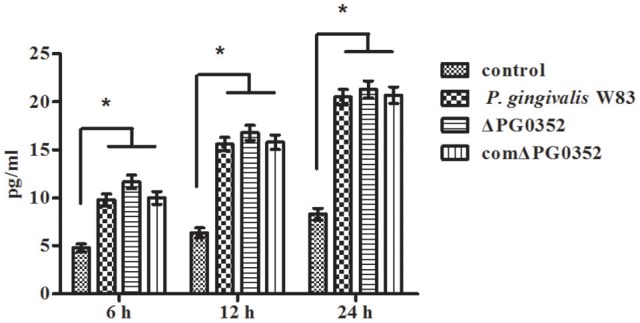
The levels of IL-12p70 in macrophages stimulated by different *P. gingivalis* strains after CR3 receptor suppressed by CD11b antibody. *P < 0.01 vs. control group, using ANOVA.

We then used anti-CD11b antibodies to suppress the CR3 receptor and detected the IL-12p70 level in each group. The level of IL-12p70 increased and showed no significant differences in the *P. gingivalis* W83, ΔPG0352 and comΔPG0352 groups after CR3 was suppressed (shown in Figure 5).

### Differential expression of miRNAs in macrophages stimulated by *P. gingivalis* W83 or ΔPG0352

**Figure 6:**
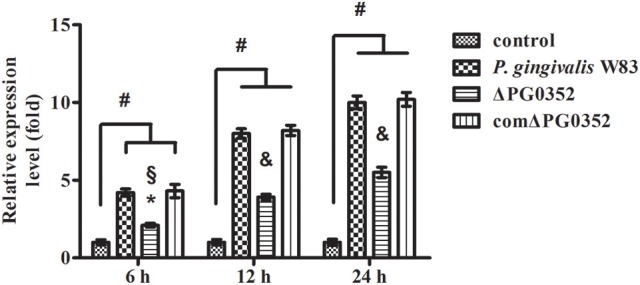
The expression of miR-21 after macrophages stimulated by *P. gingivalis* W83, ΔPG0352, or comΔPG0352. *P < 0.05 vs. control group, ^#^*P* < 0.01 vs. control group, ^§^*P* < 0.05 vs. *P. gingivalis* W83 group, ^&^*P* < 0.01 vs. *P. gingivalis* W83 group, using ANOVA.

The expression of miRNAs in macrophages in response to *P. gingivalis* stimulation was examined using a PCR array that included 84 key miRNAs predicted to regulate the expression of proinflammatory or autoimmunity genes. There were six miRNAs differently expressed between the *P. gingivalis* W83 and ΔPG0352 groups (shown in Table 1). To investigate why the level of IL-12 increased in the ΔPG0352 group, TargetScan and miRDB databases were used to forecast miRNAs correlated with IL-12. The results showed that *IL-12A* (*IL-12p35*) was the target gene of miR-21-5p and miR-590-5p, and *IL-12B* (*IL-12p40*) was the target gene of miR-23-3p and miR-130-5p. As shown in Table 1, it was found that there was a significant difference in miR-21-5p between the *P. gingivalis* W83 and ΔPG0352 groups; the fold change of expression was 0.444. The difference in miR-21 expression between the *P. gingivalis* W83 and ΔPG0352 groups was confirmed by real-time PCR for 6, 12, and 24 h (shown in Figure 6). The results showed that expression of the miR-21 gene increased gradually over time in macrophages stimulated by *P. gingivalis* and was significantly higher in U937 stimulated by *P. gingivalis* W83 than that in macrophages stimulated by ΔPG0352. These results were consistent with the results from PCR array.

**Table 1:** The significant differences of miRNA expression after macrophages stimulated by ΔPG0352 compared with *P. gingivalis* W83.

**miRNA**	**Fold change**	***P*-value**
hsa-let-7b-5p	0.313	0.009
hsa-miR-125a-5p	0.647	0.042
hsa-miR-156-5p	0.260	0.000
hsa-miR-21-5p	0.444	0.021
hsa-miR-30d-5p	0.647	0.040
hsa-miR-590-5p	0.561	0.035

### Different expression of lncRNAs in macrophages stimulated by *P. gingivalis* W83 or ΔPG0352

**Figure 7:**
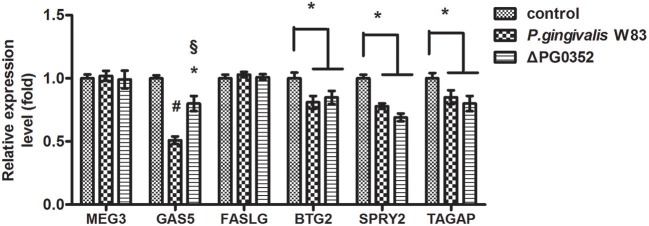
The expression of lncRNA *MEG3, GAS5, FASLG, BTG2, SPRY2*, and *TAGAP* genes after macrophages stimulated by *P. gingivalis* W83 and ΔPG0352. *P < 0.05 vs. control group, ^#^*P* < 0.01 vs. control group, ^§^*P* < 0.01 vs. *P. gingivalis* W83 group, using ANOVA.

**Figure 8:**
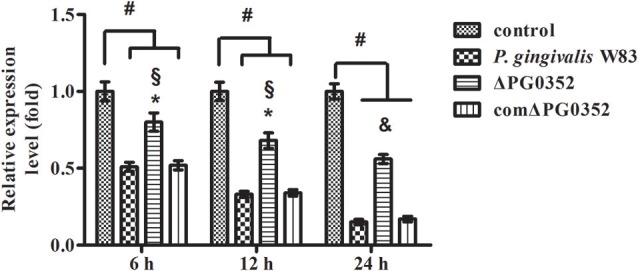
The expression of lncRNA *GAS5* after macrophages stimulated by *P. gingivalis* W83, ΔPG0352 or comΔPG0352. *P < 0.05 vs. control group, ^#^*P* < 0.01 vs. control group, ^§^*P* < 0.05 vs. *P. gingivalis* W83 group, ^&^*P* < 0.01 vs. *P. gingivalis* W83 group, using ANOVA.

According to previous findings, it was concluded that *MEG3, GAS5, FASLG, BTG2, SPRY*2, and *TAGAP* could regulate miR-21 expression. After 6 h stimulated by *P. gingivalis* W83 or ΔPG0352, the expression of *GAS5, BTG2, SPRY2*, and *TAGAP* was significantly lower in the *P. gingivalis* W83 or ΔPG0352 groups compared with the control group (shown in Figure 7). The expression of *GAS5* in the *P. gingivalis* W83 and ΔPG0352 groups was significantly different and there were no significant differences between the *P. gingivalis* W83 and ΔPG0352 groups in *MEG3, FASLG, BTG2, SPRY2*, and *TAGAP* expression. Real-time PCR was used to detect the different expression of lncRNA *GAS5* when macrophages were stimulated by different *P. gingivalis* strains for 6, 12, and 24 h. Compared to the control group, the expression of *GAS5* decreased in macrophages stimulated by *P. gingivalis* in a time dependent manner. Compared to the *P. gingivalis* W83 group, the expression of *GAS5* was significantly higher in the ΔPG0352 group (shown in Figure 8).

### The expression of *GAS5* in macrophages after transfected with lentivirus-mediated shRNA

**Figure 9:**
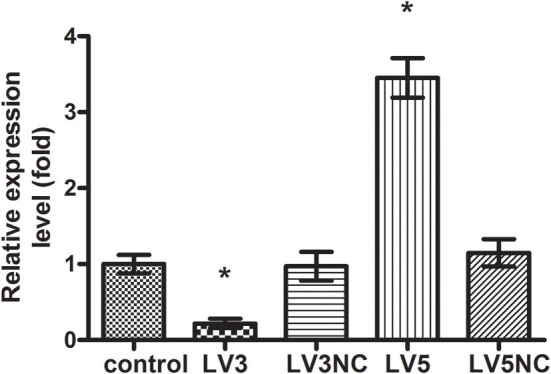
The expression of lncRNA *GAS5* genes after transfected with Lentivirus-mediated shRNA. *P < 0.05 vs. control group, using ANOVA.

The expression of *GAS5* in LV3, LV3NC, LV5, and LV5NC groups was detected by using real-time PCR (shown in Figure 9). These results indicated that LV3 significantly down-regulated the *GAS5* expression in macrophages, the silence efficiency was 78 ± 6%, and the expression of *GAS5* in LV5 group was 3.45 ± 0.26 times that of the control. These results indicated that lentivirus-mediated *GAS5* shRNA*LV3* and *LV5* were able to significantly down-regulate or up-regulate *GAS5* expression in macrophages, respectively.

### The levels of IL-12p70 in macrophages after transfected with lentivirus-mediated shRNA

**Figure 10:**
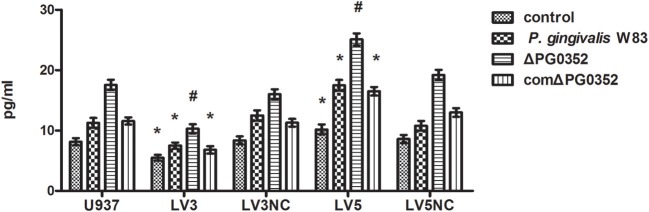
The levels of IL-12p70 after transfected with Lentivirus-mediated shRNA. *P < 0.05 vs. control group, ^#^*P* < 0.01 vs. control group, using ANOVA.

ELISA was used to detect the level of IL-12p70 in transfected macrophages stimulated by *P. gingivalis* W83, ΔPG0352 or comΔPG0352 after 24 h (shown in Figure 10). The results showed that, compared with U937, the levels of IL-12p70 were significantly lower in *LV3*, and higher in *LV5* after stimulated by different *P. gingivalis* strains. The differences of IL-12 level between the *P. gingivalis* W83 and ΔPG0352 groups in *LV3* were less than those in U937.

### The difference in *GAS5* expression between *P. gingivalis* W83 and ΔPG0352 groups was associated with CR3

**Figure 11:**
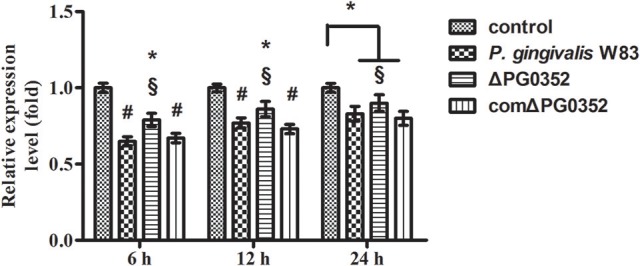
The expression of lncRNA *GAS5* in macrophages stimulated by *P. gingivalis* W83, ΔPG0352 or comΔPG0352 after CR3 suppressed by CD11b antibody. *P < 0.05 vs. control group, ^#^*P* < 0.01 vs. control group, ^§^*P* < 0.05 vs. *P. gingivalis* W83 group, using ANOVA.

The expression of *GAS5* increased in each group after CR3 receptor was suppressed by anti-CD11b antibodies (shown in Figure 11). Compared with the *P. gingivalis* W83 and comΔPG0352 groups, *GAS5* expression was significantly higher in the ΔPG0352 group from 6 to 24 h (*P* < 0.01). There were no significant differences between the *P. gingivalis* W83 and comΔPG0352 groups in the gene expression of *GAS5* (*P* > 0.05).

## Discussion

Macrophages are an important part of the innate immune system. They can engulf pathogens, regulate immune function by antigen presentation, and secreting inflammatory cytokines to clear pathogens invading host tissues. The phagocytosis of macrophages includes the recognition of surface receptors of pathogens, wrapping pathogens and clearing them. In this study, we stimulated U937 with *P. gingivalis* and performed TEM assays to observe the appearance of *P. gingivalis* internalized into macrophages. The results showed that there was microvilli formation on the macrophage surface and pits surrounding the pathogens. *P. gingivalis* W83, ΔPG0352 and comΔPG0352 were visible within the cytoplasm of macrophages, enclosed within membrane-bound endocytic vacuoles. Therefore, we suggest that irrespective of sialidase deficiency, *P. gingivalis* can interact with macrophages inducing phagocytes is to clear pathogens to different extents.

The inflammatory response is essential for host defense against invading pathogens. Previous studies showed that IL-12 produced by macrophages plays an important role in resistance to pathogen infection (Trinchieri, 2003). IL-12 is described as a natural killer cell stimulatory factor (NKSF), linking the activation of innate immune cells to the induction of an effective adaptive immune response (Rossol et al., 2011). The results in this study showed that both the expression of *IL-12p35* and *IL-12p40* genes and the level of IL-12p70 increased in U937 stimulated by both *P. gingivalis* W83 and ΔPG0352. The expression of IL-12 was significantly higher in U937 stimulated by ΔPG0352 than *P. gingivalis* W83, indicating that sialidase deficiency in *P. gingivalis* W83 increases IL-12 levels in macrophages. There are two possible reasons for this observation. The first reason might be that the lack of sialidase reduces the activity of important *P. gingivalis* virulence factors, such as fimbriae, LPS, capsule, and gingipains (Aruni et al., 2011; Li et al., 2012, 2017). Virulence factors of *P. gingivalis* affect the interaction with macrophages. Gmiterek et al. (2016) found that the HmuY gene mutant in *P. gingivalis* affected the interaction between *P. gingivalis* and macrophages by decreasing cytokines and chemokines. Singh et al. compared the host inflammatory response induced by encapsulated and non-encapsulated *P. gingivalis* and found that the capsule of *P. gingivalis* reduced the host inflammatory response (Singh et al., 2011). Hou et al. showed that *P. gingivalis* gingipain decreased expression of IL-12 which *Escherichia coli* LPS induced in macrophages (Hou et al., 2017). The second reason might be that the macromolecules on the cell surface of ΔPG0352 cannot be sialylated due to sialidase deficiency. This would affect the interaction between *P. gingivalis* and macrophages.

Several publications reported data about IL-12 expression in macrophages stimulated by whole *P. gingivalis* cells. Sugano et al. and Huang et al. found that the expression of IL-12 induction in macrophages stimulated by *P. gingivalis* was two-fold higher than that of controls at 24 h post-infection (Sugano et al., 2004; Huang et al., 2011). These results are consistent with the data presented here. Conversely, Park et al. found that *P. gingivalis* LPS can increase the level of IL-12 in macrophages nine-fold (Park et al., 2011). These results demonstrated that some components in *P. gingivalis*, such as LPS, can increase the level of IL-12 in macrophages and some may attenuate this increase. Besides, different *P. gingivalis* strains induced different levels of IL-12 in macrophages. Gmiterek et al. (2016) stimulated U937 with *P. gingivalis* A7436, the levels of IL-12 increased much higher than that stimulated by *P. gingivalis* W83 the data presented here. Sugano et al. also compared cytokine induction in two strains of *P. gingivalis*, and the results showed that *P. gingivalis* ATCC 49417 showed statistically higher gene expression of IL-12 induction than that of controls at 24 h post-infection. However, *P. gingivalis* 381 showed no significant induction of IL-12 levels (Sugano et al., 2004). These data indicate that different *P. gingivalis* strains can result in different IL-12 expression levels in macrophages.

Complement receptor 3 (CR3; CD11b/CD18), which is the most common integrin expressed abundantly in monocytes and neutrophils (Bhat et al., 1999; Yakubenko et al., 2002), can recognize and interact with molecules derived from host cells and pathogens. The interaction of CD11b/CD18 and *P. gingivalis* is very important for *P. gingivalis* to internalize and subvert phagocytosis by macrophages (Hajishengallis et al., 2007). The immunofluorescence results presented here showed that the expression of CR3 was significantly lower than that of *P. gingivalis* W83 and comΔPG0352 groups, indicating that sialidase deficiency attenuated the ability of *P. gingivalis* activating CR3 in macrophages. There are three possible reasons for this observation. First, sialidase deficiency influences the synthesis and modification of the macromolecules on *P. gingivalis* surface, which affect *P. gingivalis* to bind with macrophages and induce less CR3 expression. Second, sialidase deficient mutant strain fails to produce an intact capsule. Capsule contains antigen, immature capsule in sialidase deficient mutant strain cannot activate CR3 as much as mature capsule in wild-type strain. Third, deletion of sialidase gene in *P. gingivalis* W83 reduces gingipain activity and the amount of LPS, thus sialidase deficient mutant strain induces less CR3 expression than wild type. CR3 was reported to regulate production of inflammatory mediators and phagocytosis of cells by inside-out signaling pathways (Ehlers, 2000; Laudanna et al., 2002). When *P. gingivalis* interacts with CR3 on the surface of macrophages, extracellular signal-regulated kinases (ERK) 1/2 is activated (Trinchieri, 2003). The activation of ERK1/2 reduced the level of IL-12p70, and inhibited IL-12 mediated clearance to pathogens (Trinchieri, 2003). Therefore, CR3 is thought to be exploited by *P. gingivalis* to undermine IL-12-mediated bacterial clearance (Hajishengallis et al., 2007). Harokopakis and Shimaoka inhibited CR3 in a mouse periodontitis model and the results showed that the survivability of *P. gingivalis* in host cells was reduced, thus inhibiting alveolar bone resorption (Shimaoka et al., 2002; Harokopakis et al., 2006). In this study, the levels of IL-12p70 became higher in macrophages stimulated by *P. gingivalis* and much higher in the ΔPG0352 group than in the *P. gingivalis* W83 and comΔPG0352 groups. There were no significant differences between the *P. gingivalis* W83, ΔPG0352 and comΔPG0352 groups when CR3 was suppressed by anti-CD11b. These results indicated that sialidase deficiency in *P. gingivalis* weakened the increasing level of CR3 in macrophages. Compared with *P. gingivalis* W83, ΔPG0352 could stimulate more IL-12 secretion in macrophages. The difference in IL-12 levels between the *P. gingivalis* W83 and ΔPG0352 groups were associated with CR3.

In order to explore why sialidase-deficient *P. gingivalis* induced more IL-12 in macrophages, microRNA PCR array was carried out. It was shown that miR-21, which could regulate the expression of IL-12, was significantly lower in macrophages stimulated by ΔPG0352. MiR-21 is one of the most highly expressed microRNAs in multiple types of mammalian cells (Lagos-Quintana et al., 2001, 2002; Landgraf et al., 2007). In addition, MiR-21 is also expressed in immune cells, including monocytes, macrophages, B/T-cells and dendritic cells (DCs) (Sheedy, 2015). Previous studies have shown that miR-21 is up-regulated in LPS-treated periodontal ligament cells (Du et al., 2016) and also in various cancers (Wang et al., 2014). Finally, miR-21 levels are thought to be a marker of immune cell activation (Kasashima et al., 2004; Landgraf et al., 2007). Up-regulated miR-21 in macrophages/monocytes is commonly related to pro-inflammatory factors by viruses, bacteria and other molecular patterns and plays an important role in the innate immune process (Sheedy, 2015). TargetScan and miRDB database analysis showed that miR-21 could negatively regulate the expression of IL-12. Lu et al. constructed a luciferase receptor vector to determine the direct effect of miR-21 on *IL-12p35* (Lu et al., 2009). The results indicated that *IL-12p35* is a target gene of miR-21; moreover, miR-21 has the 8-mer seed sequence that binds to the *IL-12p35* 3'UTR region, resulting in the repression of IL-12. Therefore, the sialidase-deficient strain of *P. gingivalis* secreted more IL-12 by inhibiting the level of miR-21 in macrophages.

LncRNAs are defined as a kind of long-chain RNA with transcripts longer than 200 bp. These molecules are involved in regulation of gene expression directly by participating in protein modifications or recruitment of transcription factors. In this study, we also screened six lncRNAs associated with the expression of miR-21 by referring to previous reports. Among these lncRNAs, only growth arrest-specific 5 (*GAS5*) showed significantly different levels between macrophages stimulated by *P. gingivalis* W83 and ΔPG0352. *GAS5*, which has an identified gene regulatory function, was originally found to accumulate in growth-arrested cells (Schneider et al., 1988). *GAS5* levels are alerted in virus or bacterial infectious diseases. Mayama et al. found that *GAS5* played an important roles in the regulation of immune functions and pathogenesis/pathophysiology of autoimmune, inflammatory, and infectious diseases (Mayama et al., 2016). The results in this study showed that the expression of *GAS5* decreased in macrophages stimulated by *P. gingivalis*. However, compared to the *P. gingivalis* W83 group, the expression of *GAS5* was significantly higher in macrophages stimulated by ΔPG0352. The levels of IL-12p70 were lower when *GAS5* was silenced in macrophages and higher when *GAS5* was overexpressed. Differences in IL-12p70 levels between *P. gingivalis* W83 and ΔPG0352 groups were reduced when *GAS5* was silenced in macrophages. Therefore, compared to *P. gingivalis* W83, the sialidase-deficient strain of *P. gingivalis* reduced the inhibition of *GAS5* leading to more IL-12 secretion in macrophages. Recently, reports have shown that *GAS5* is a direct target of miR-21 through interaction with the putative miR-21-binding site at exon 4 of *GAG5*, which indicates that *GAS5* negatively regulates miR-21 through the RNA-induced silencing complex (RISC) (Zhang et al., 2013; Song et al., 2014). Taken together, these results indicated that compared to *P. gingivalis* W83, the sialidase-deficient strain of *P. gingivalis* reduced the inhibition of *GAS5*, induced lower expression of miR-21, and led to higher IL-12 level in macrophages.

To our knowledge, there have been few reports associated with the relationship between CR3 and lncRNA *GAS5*. To explore the relationship between CR3 and lncRNA *GAS5*, we used anti-CD11b antibodies to suppress CR3 and detected the expression of *GAS5* by real-time PCR. The results showed that *GAS5* expression increased after CR3 suppression in all three *P. gingivalis* groups. *GAS5* was higher in the ΔPG0352 group compared with the *P. gingivalis* W83 and comΔPG0352 groups. This result indicated a reciprocal interaction between CR3 and *GAS5*. It was noteworthy that the level of IL-12p70 showed no significant differences in *P. gingivalis* W83, ΔPG0352, and comΔPG0352 groups after CR3 was suppressed. However, there were differences in the IL-12p70 levels in the ΔPG0352 and the *P. gingivalis* W83 groups after silencing *GAS5*. We speculated the reason was that, in addition to *GAS5*, there was another pathway which could take part in the influence of CR3 on IL-12 production.

**Figure 12:**
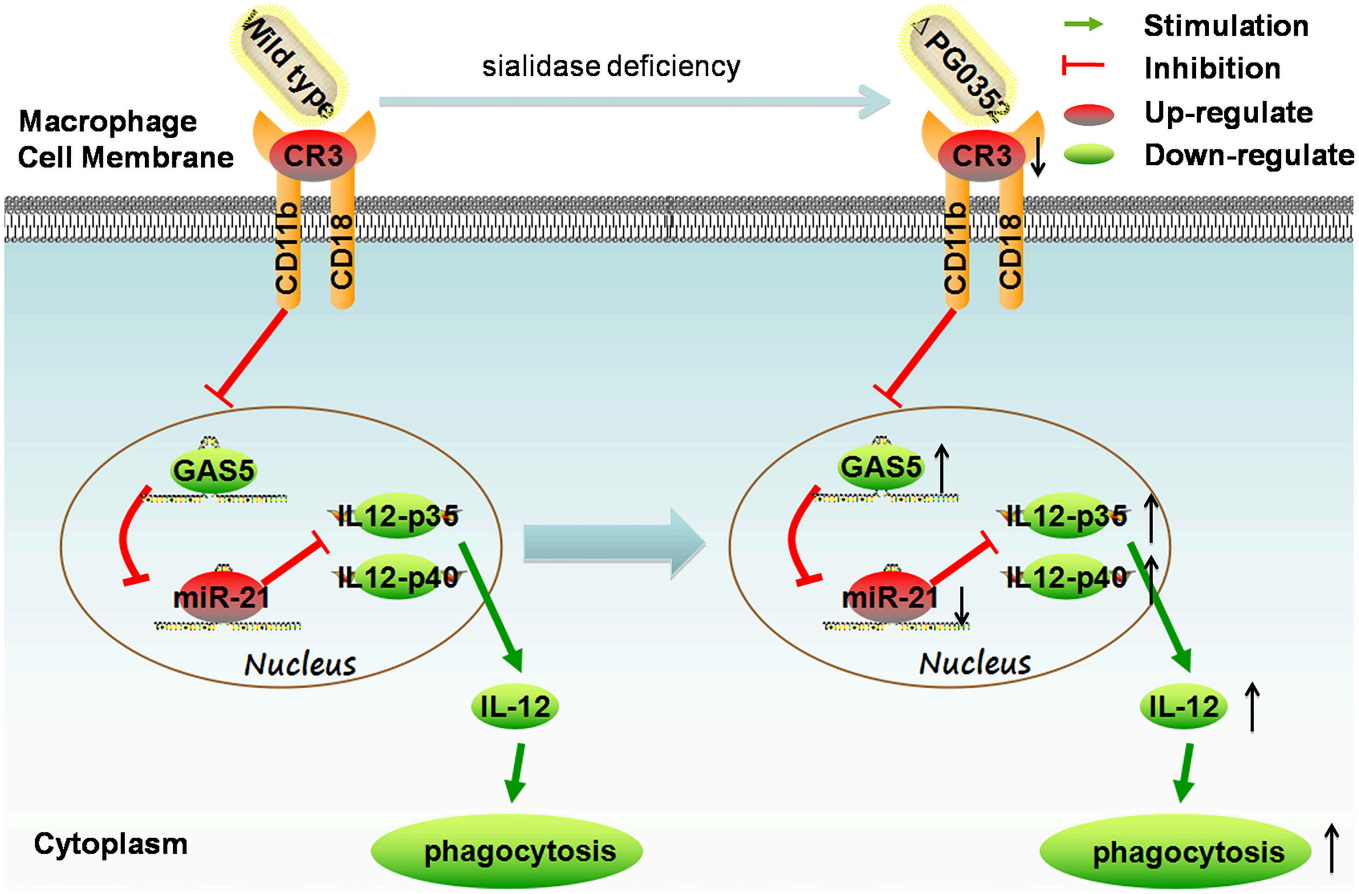
The mechanism of sialidase deficiency increasing IL-12 secretion in *P. gingivalis*-stimulated macrophages. *P. gingivalis* can activate CR3 in macrophages, inhibit the expression of lncRNA GAS5, increase the expression of miR-21, decrease the level of IL-12 and subvert phagocytosis by macrophages. The sialidase-deficiency in *P. gingivalis* attenuates CR3 activation in macrophages, reduces the inhibition of lncRNA *GAS5*, induces less miR-21 and more IL-12 in macrophages.

In this study, we first found that compared to *P. gingivalis* W83, sialidase deficiency in *P. gingivalis* induced more IL-12 in macrophages. Second, the mechanism was clarified by using PCR array, gene silence and overexpression experiments. It can be concluded that the sialidase-deficiency in *P. gingivalis* attenuates CR3 activation in macrophages, reduces the inhibition of lncRNA *GAS5*, induces less miR-21 and more IL-12 in macrophages. (shown in Figure 12). The results of this study indicate that inhibiting the activity of sialidase in *P. gingivalis* would render *P. gingivalis* more easily cleared by macrophages. This finding will open up a new direction in prevention and treatment of chronic periodontitis.

## Author contributions

CL, YP, and JL conceived and designed the experiments. XY, XX, TT, SY, LL, and YZ performed the experiments. YP, LL, and DZ analyzed the data. XY, CL, and YP wrote the manuscript.

### Conflict of interest statement

The authors declare that the research was conducted in the absence of any commercial or financial relationships that could be construed as a potential conflict of interest.

## Supplementary material

Image 1 Map of LV3 lentiviral vector (H1/GFP&Puro).

Image 2 Map for LV5 lentiviral vector (EF-1α/GFFP&Puro).
