# Supplementary figures and images for: Sialidase Deficiency in Porphyromonas gingivalis Increases IL-12 Secretion in Stimulated Macrophages Through Regulation of CR3, IncRNA GAS5 and miR-21
Source: Front Cell Infect Microbiol. 2018 Apr 5;8:100. doi: 10.3389/fcimb.2018.00100 (PMC5895773; doi:10.3389/fcimb.2018.00100)

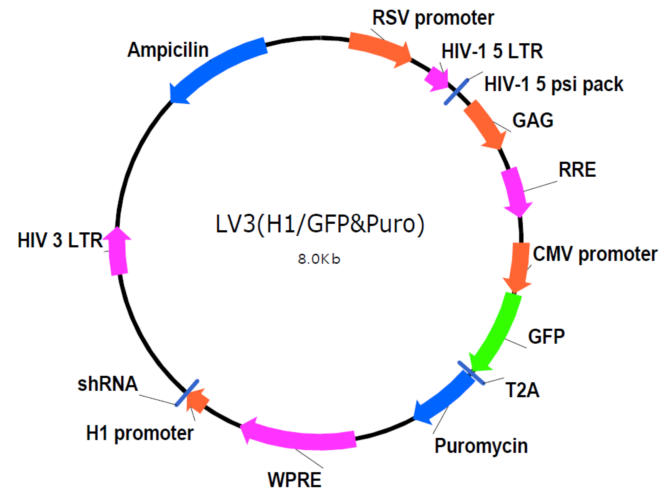

Supplement: Image 1 — Map of LV3 lentiviral vector (H1/GFP&Puro). [file Image1.TIF]

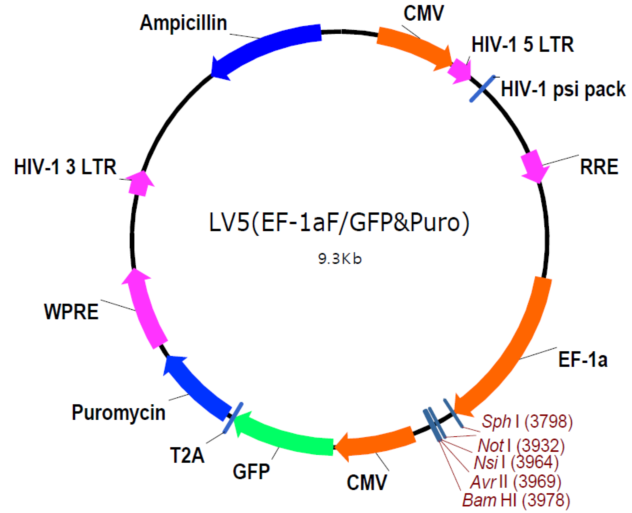

Supplement: Image 2 — Map for LV5 lentiviral vector (EF-1α/GFFP&Puro). [file Image2.TIF]
